# Cell membrane disruption stimulates cAMP and Ca^2+^ signaling to potentiate cell membrane resealing in neighboring cells

**DOI:** 10.1242/bio.028977

**Published:** 2017-11-01

**Authors:** Tatsuru Togo

**Affiliations:** Department of Anatomy, St. Marianna University School of Medicine, 2-16-1 Sugao, Miyamae, Kawasaki, Kanagawa 216-8511, Japan

**Keywords:** Membrane resealing, ATP, cAMP, Ca^2+^, Protein kinase A, Protein kinase C

## Abstract

Disruption of cellular plasma membranes is a common event in many animal tissues, and the membranes are usually rapidly resealed. Moreover, repeated membrane disruptions within a single cell reseal faster than the initial wound in a protein kinase A (PKA)- and protein kinase C (PKC)-dependent manner. In addition to wounded cells, recent studies have demonstrated that wounding of Madin-Darby canine kidney (MDCK) cells potentiates membrane resealing in neighboring cells in the short-term by purinergic signaling, and in the long-term by nitric oxide/protein kinase G signaling. In the present study, real-time imaging showed that cell membrane disruption stimulated cAMP synthesis and Ca^2+^ mobilization from intracellular stores by purinergic signaling in neighboring MDCK cells. Furthermore, inhibition of PKA and PKC suppressed the ATP-mediated short-term potentiation of membrane resealing in neighboring cells. These results suggest that cell membrane disruption stimulates PKA and PKC via purinergic signaling to potentiate cell membrane resealing in neighboring MDCK cells.

## Abstract (precis)

**Summary:** Real-time imaging showed that cell membrane disruption induced cAMP and Ca^2+^ signaling in neighboring MDCK cells. These responses were required for faster cell membrane repair in neighboring cells.

## INTRODUCTION

Mechanical stress induces cell membrane disruption in many animal tissues under physiological conditions (McNeil and Steinhardt, 2003). Typically, these disruptions are rapidly resealed by exocytic and endocytic mechanisms triggered by the influx of extracellular Ca^2+^ (Blazek et al., 2015).

Cell membrane disruption stimulates various signaling pathways so that repeated membrane disruptions within a single cell reseal faster than the initial wound (Togo et al., 1999, 2003; Togo, 2004; Shen and Steinhardt, 2005). These potentiated responses depend on protein kinase A (PKA) and protein kinase C (PKC) in the early stages (Togo et al., 1999, 2003; Shen and Steinhardt, 2005). In the long-term (24 h), potentiation of membrane resealing in wounded cells depends on cAMP response element (CRE)-mediated gene expression via a PKC- and p38 mitogen-activated protein kinase-dependent pathway (Togo et al., 2003; Togo, 2004).

In addition to wounded cells, injury of Madin-Darby canine kidney (MDCK) cells has been shown to potentiate membrane resealing in neighboring cells (Togo, 2012, 2014). Long-term responses require nitric oxide/protein kinase G signaling to stimulate CRE-mediated gene expression in neighboring cells (Togo, 2012). However, in the short-term, potentiation of membrane resealing in neighboring cells depends on purinergic signaling mediated by ATP (Togo, 2014). It has been proposed that these multicellular adaptive responses are able to efficiently protect tissues from mechanical stresses (Togo et al., 2003; Togo, 2012, 2014). This is important as animals normally generate and are exposed to repetitive mechanical stresses, and these stresses are thought to result in cell membrane disruptions in many cases (McNeil and Steinhardt, 2003).

The aim of the present study was to investigate downstream signaling pathways stimulated by ATP in neighboring MDCK cells. Fluorescence live cell imaging showed that purinergic signaling stimulated cAMP synthesis and Ca^2+^ mobilization in neighboring cells. Furthermore, membrane resealing assays revealed the involvement of PKA and PKC in the short-term potentiation of membrane resealing in neighboring cells in addition to wounded cells.

## RESULTS

### Wounding stimulates cAMP synthesis and Ca^2+^ mobilization in neighboring MDCK cells

**Fig. 1.**
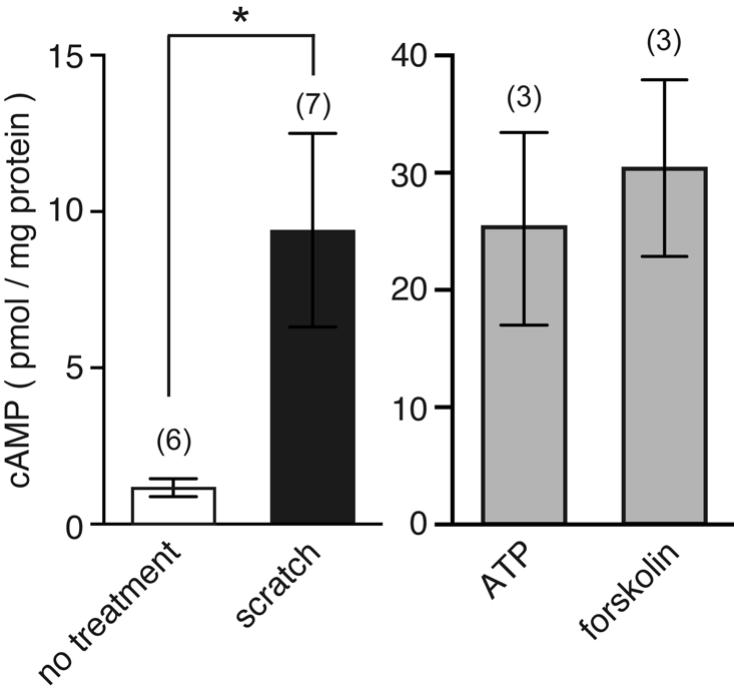
**Scratching monolayers stimulates cAMP synthesis in MDCK cells.** Confluent monolayers of MDCK cells were maintained in 1.8 mM Ca^2+^ Ringer's solution containing 100 µM IBMX, a phosphodiesterase inhibitor. Cells were then wounded by scratching monolayers with a 27G needle 20 times, or treated with 100 µM ATP or 100 µM forskolin. The amount of cAMP was quantified by immunoassay and normalized to the amount of total protein. The number of experiments is indicated in parentheses. *P=0.0006.

To determine whether cell membrane disruption can stimulate cAMP production, monolayers of MDCK cells were injured by slowly scratching them 20 times with a 27G needle. Scratching monolayers induces cell membrane disruption along the scratched line (Grembowicz et al., 1999; Togo, 2012). After scratching, the amount of cAMP was quantified by an immunoassay (Fig. 1). Scratching monolayers stimulated cAMP production (9.4±3.1 pmol/mg protein, *n*=7). For controls, cells were treated with either 100 µM ATP or 100 µM forskolin, an activator of adenylyl cyclase (AC) (Fig. 1). As expected, these treatments also induced cAMP production. The amount of cAMP produced was much higher than that of scratched monolayers, as ATP and forskolin stimulated all the cells in the well.

**Fig. 2.**
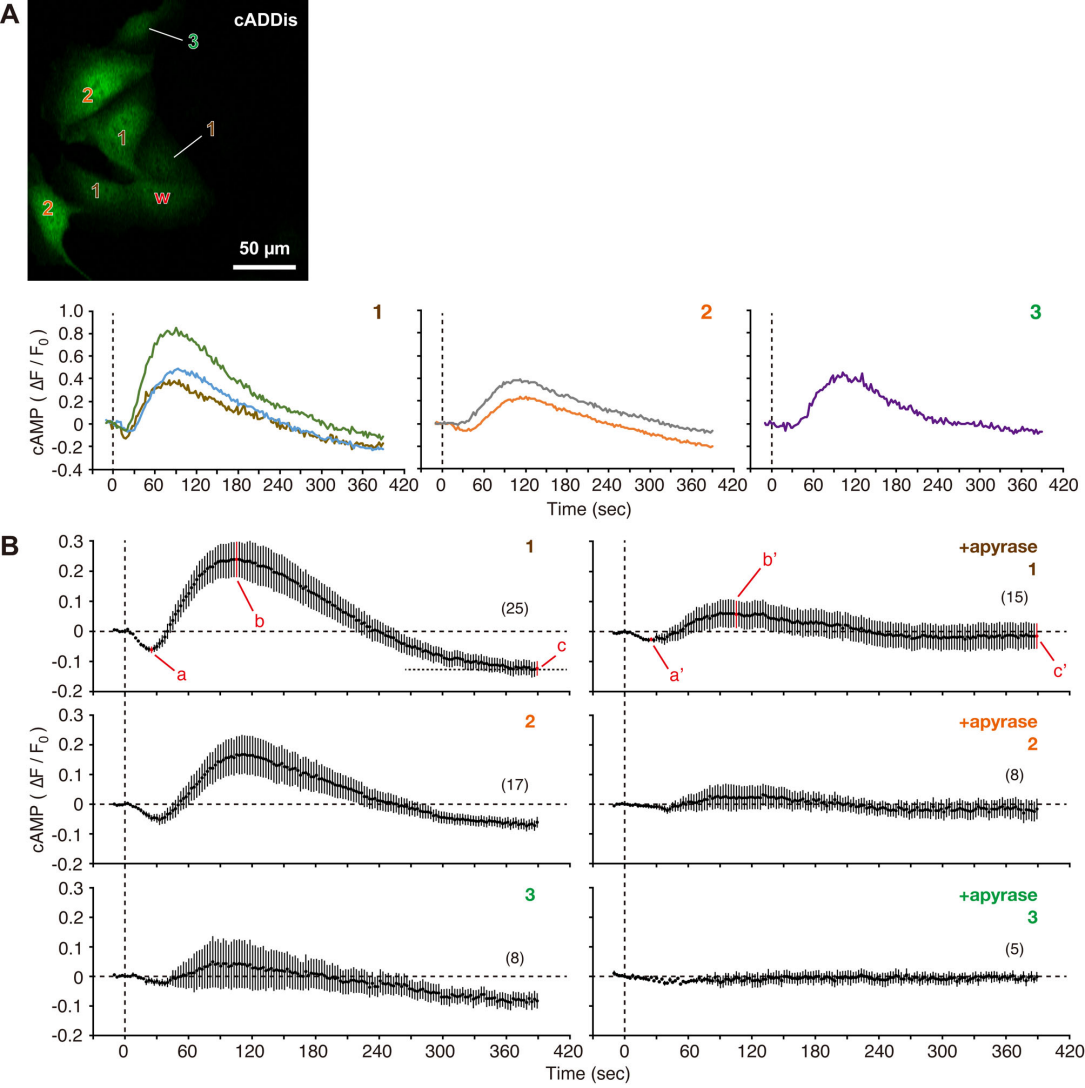
**Cell membrane disruption stimulates cAMP production in neighboring cells.** (A) ‘w’ in the fluorescence image of MDCK cells expressing Green Upward cADDis indicates a wounded cell. Cells adjacent to the wounded cell were labeled with numbers in order of their proximity to the wounded cell. A cell was wounded at time zero with a glass needle in 1.8 mM Ca^2+^ Ringer's solution, and the time course of changes in fluorescence intensity of cADDis was plotted for neighboring cells (1–3). The image shown in this figure was acquired 90 s after cell membrane disruption. See also Movie 1. (B) Cells were wounded at time zero with a glass needle in the absence or presence of 20 U/ml apyrase, and changes in fluorescence intensity in neighboring cells were compared. The number of observed cells is indicated in parentheses. *P*=0.0007 (a–a′); *P*=0.0427 (b–b′); *P*=0.0197 (c–c′).

Although scratching monolayers clearly demonstrated cAMP synthesis upon wounding, it was impossible to determine the spatiotemporal dynamics of cAMP signaling by this assay. Thus, cAMP Difference Detector *in situ* (cADDis) was used to analyze the synthesis of cAMP after cell membrane disruption (Fig. 2). cADDis is a green fluorescent protein that changes its fluorescence intensity in response to an increase in cAMP (Tewson et al., 2016). When MDCK cells expressing Green Upward cADDis were wounded with a glass needle, the cADDis fluorescence intensity in neighboring cells initially decreased (Fig. 2B,a) and then increased transiently (Fig. 2B,b). Furthermore, the baseline cADDis value in neighboring cells decreased after cell membrane disruption (Fig. 2B,c), compared with the initial value. The changes in the cADDis fluorescence intensity gradually decreased with increased distance from the wounded cells.

Inhibition of purinergic signaling by 20 U/ml apyrase significantly attenuated the cAMP signaling in neighboring cells (Fig. 2B,a′ and b′). Furthermore, the baseline cADDis intensity did not decrease after cell membrane disruption in the presence of apyrase (Fig. 2B,c′).

**Fig. 3.**
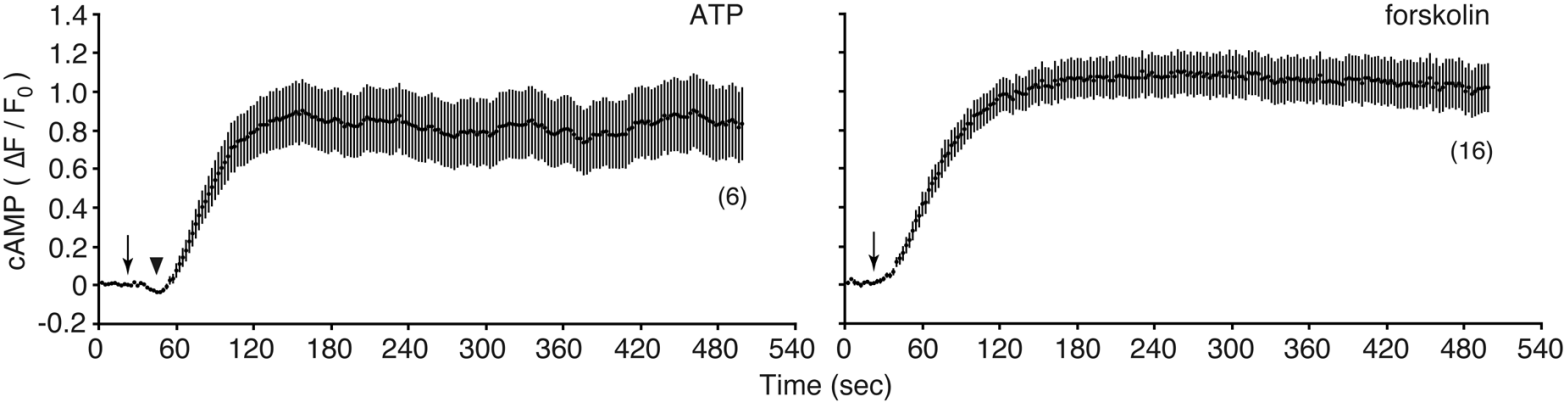
**ATP and forskolin stimulate cAMP synthesis in MDCK cells.** Cells expressing Green Upward cADDis were treated with either 100 µM ATP or 100 µM forskolin at the time indicated by arrows, and the changes in fluorescence intensity of cADDis were recorded. The arrowhead indicates the transient decrease in fluorescence intensity. The number of observed cells is indicated in parentheses.

Treatment of cells with 100 µM ATP induced a transient decrease (indicated by an arrowhead in Fig. 3), followed by an increase in the fluorescence intensity of cADDis, as observed in cells adjacent to wounded cells. Direct stimulation of AC by 100 µM forskolin induced an increase in the fluorescence intensity of cADDis, although the initial transient decrease in fluorescence intensity was not observed (Fig. 3). These results indicate that cell membrane disruption stimulates the synthesis of cAMP in neighboring cells via purinergic signaling.

**Fig. 4.**
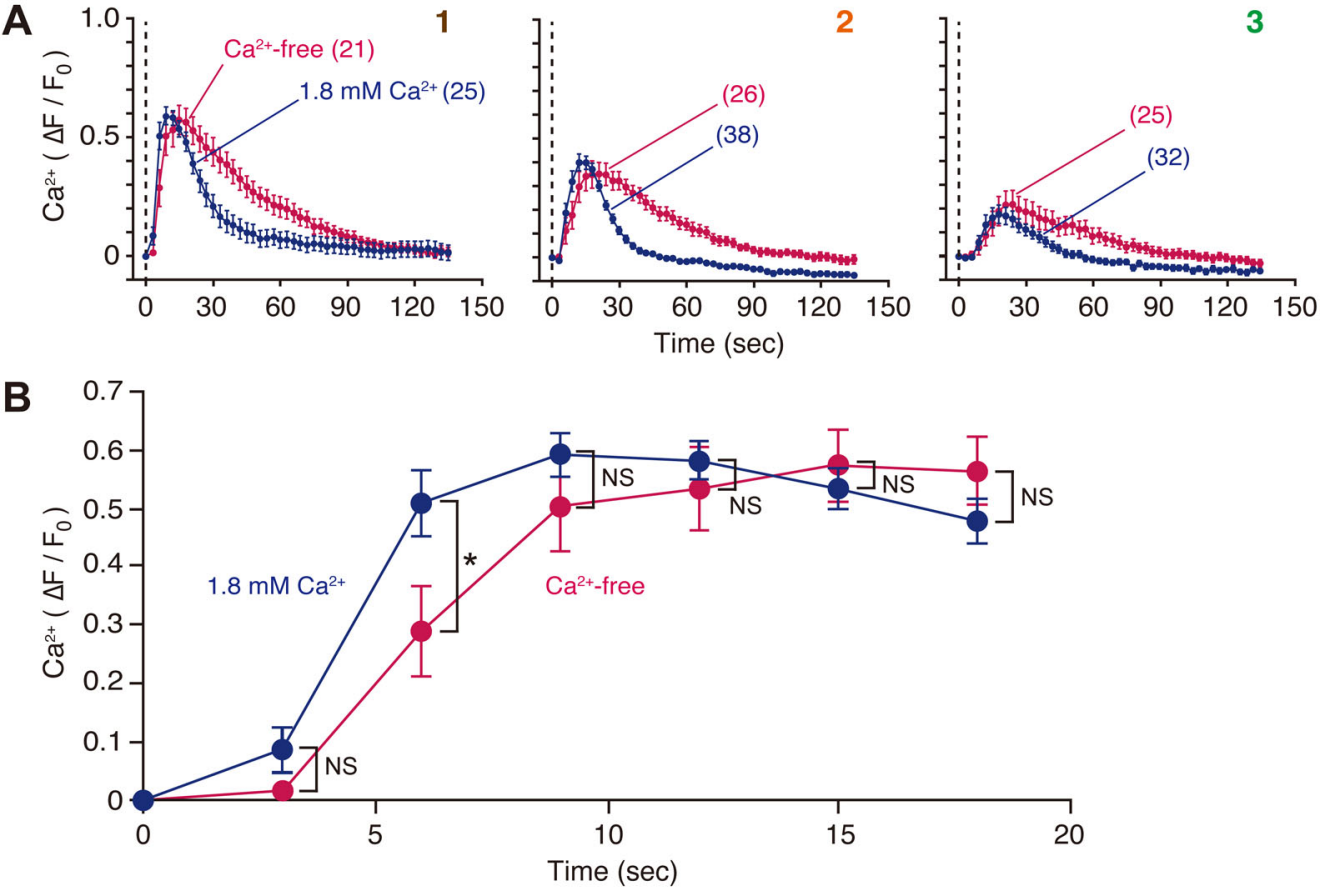
**Cell membrane disruption induces Ca^2+^ mobilization in neighboring MDCK cells.** (A) Cells loaded with Calcium Green-1 AM were wounded at time zero with a glass needle in the presence or absence of extracellular Ca^2+^, and changes in fluorescence intensity in the cytoplasmic region were compared. Cells were numbered as per Fig. 2A. The number of observed cells is indicated in parentheses. See also Movie 2. (B) To compare the initial phase of increase in [Ca^2+^]_i_, data from cell #1 in A were expanded. *P=0.0333; NS, not significant.

A previous study has demonstrated that cell membrane disruption induced intercellular Ca^2+^ signaling, which was mediated by ATP (Togo, 2014). To determine whether the increase in intracellular Ca^2+^ concentration ([Ca^2+^]_i_) in neighboring cells was due to mobilization of intracellular stores or influx from the extracellular milieu, cells loaded with Calcium Green-1 (CG-1) acetoxymethyl (AM) ester (1 µM) were wounded with a glass needle, and changes in fluorescence intensity in the cytoplasmic region upon cell membrane disruption were examined in the presence or absence of extracellular Ca^2+^ (Fig. 4A). Increase in [Ca^2+^]_i_ in neighboring cells was observed in both conditions. The peak ΔF/F_0_ values were statistically independent of external Ca^2+^, although increases in [Ca^2+^]_i_ were slightly delayed under Ca^2+^-free conditions (Fig. 4B). Furthermore, the increase in [Ca^2+^]_i_ under Ca^2+^-free conditions was prolonged compared with conditions containing 1.8 mM Ca^2+^ (Fig. 4A).

### Cell membrane disruption potentiates membrane resealing in neighboring cells in a PKA- and PKC-dependent manner

**Fig. 5.**
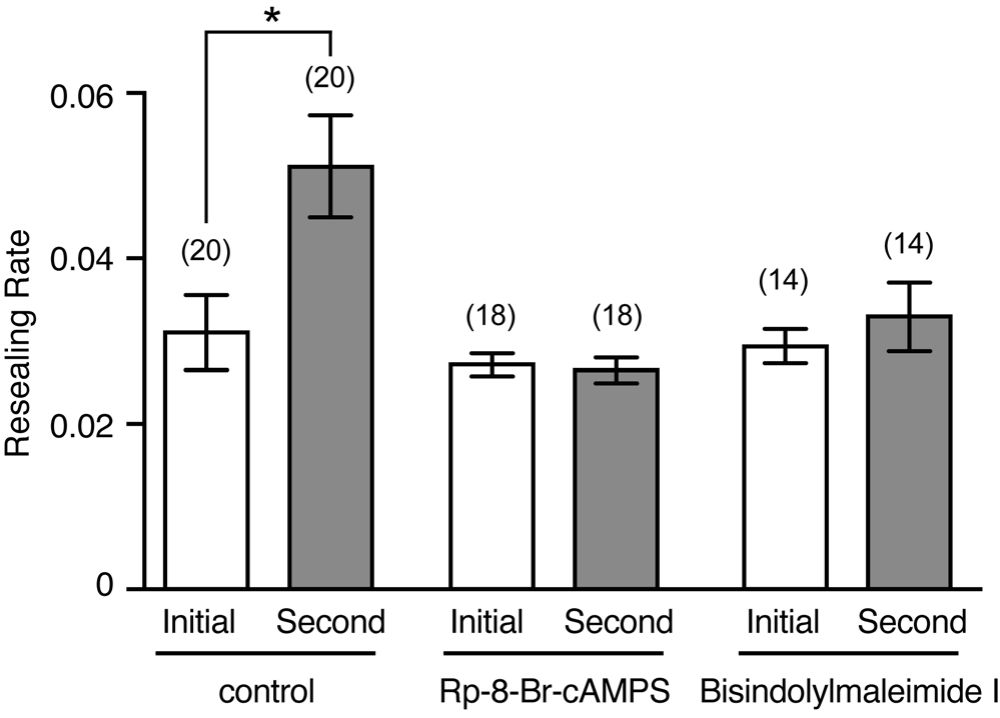
**Cell membrane disruption potentiates membrane resealing in neighboring cells in a PKA- and PKC-dependent manner.** Cells loaded with Calcein Red-Orange AM were initially wounded with a glass needle, and changes in fluorescence intensity of Calcein Red-Orange were monitored. Neighboring cells were wounded 5 min later and changes in fluorescence intensity were recorded. The resealing rate was defined as the reciprocal of resealing time in seconds. For cells that failed to reseal, the rate was defined as zero. The number of observed cells is indicated in parentheses. *P=0.002.

To analyze the short-term potentiation of cell membrane resealing in neighboring cells, MDCK cells loaded with Calcein Red-Orange AM (1 µM) were wounded using a glass needle in 1.8 mM Ca^2+^ Ringer's solution, and the changes in the fluorescence intensity of Calcein Red-Orange were monitored. To compare the timing of membrane resealing between the initial and second wounds created in neighboring cells (adjacent to initially wounded cells), the resealing time (duration of the decrease in fluorescence intensity resulting from dye efflux) was measured (Togo, 2012, 2014). The reciprocal of resealing time was termed the ‘resealing rate’, as previously reported (Steinhardt et al., 1994; Togo et al., 1999, 2000, 2003; Togo, 2004, 2006, 2012, 2014; Togo and Steinhardt, 2004; Shen and Steinhardt, 2005). When non-wounded MDCK cells were initially wounded in 1.8 mM Ca^2+^ Ringer's solution, the resealing rate was 0.031±0.0045 (*n*=20; Fig. 5). When neighboring cells were wounded 5 min after the initial wound, the resealing rate of the neighboring cells significantly increased (*P*=0.002) to 0.051±0.0062 (*n*=20) (Fig. 5). Consistent with a previous study (Togo, 2014), these results indicated that membrane resealing of neighboring MDCK cells is potentiated within 5 min. To investigate the involvement of PKA and PKC in the short-term potentiation of membrane resealing in neighboring cells, cells were pretreated for 10 min with either Rp-8-Br-cAMPS (30 µM), a membrane permeable PKA inhibitor, or bisindolylmaleimide I (1 µM), a membrane permeable PKC inhibitor, before being wounded with a glass needle. When non-wounded cells were initially wounded in the presence of Rp-8-Br-cAMPS or bisindolylmaleimide I, the membrane resealing was not affected by these treatments, as the resealing rates were 0.0027±0.0041 (*n*=18) and 0.029±0.0014 (*n*=14), respectively (Fig. 5). When neighboring cells were wounded 5 min after the initial wound, the resealing rate of the neighboring cells was 0.0026±0.0016 (*n*=18) or 0.033±0.0041 (*n*=14), respectively (Fig. 5). These results indicate that Rp-8-Br-cAMPS and bisindolylmaleimide I suppressed the short-term potentiation of membrane resealing in neighboring cells.

**Fig. 6.**
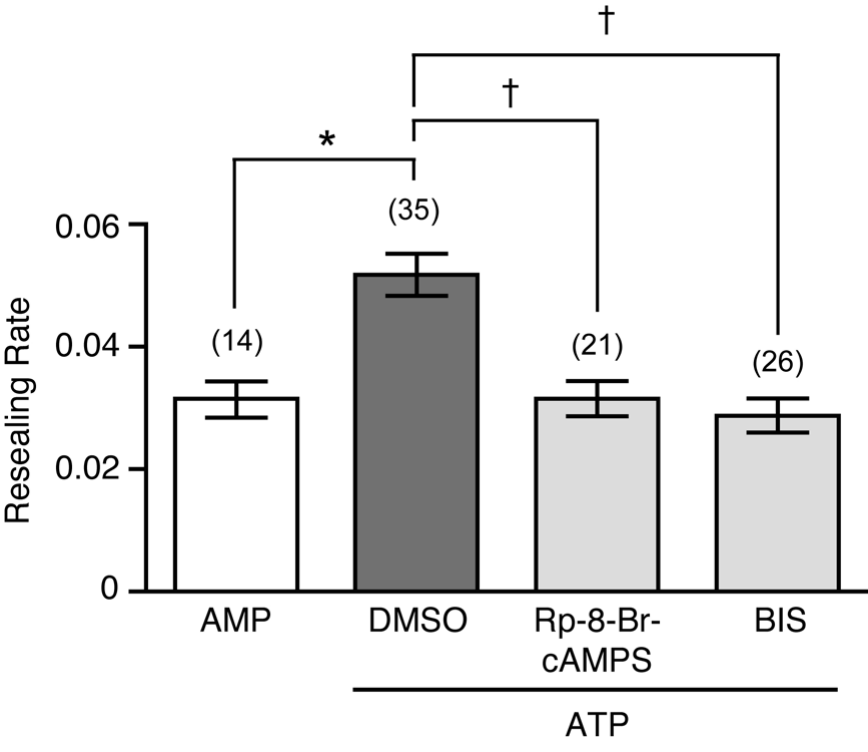
**Potentiation of membrane resealing induced by ATP is suppressed by PKA and PKC inhibitors in MDCK cells.** Cells were pretreated for 10 min with either kinase inhibitors or DMSO. Then, cells were wounded with a glass needle after the addition of 100 µM ATP in the presence or absence of inhibitors, respectively, and the resulting resealing rates were analyzed. As a control, cells treated with 100 µM AMP were wounded with a glass needle. Resealing rates were analyzed 5–20 min after the addition of nucleotides. The number of observed cells is indicated in parentheses. *P=0.0007; †*P*<0.0001.

To confirm the involvement of ATP-stimulated PKA and PKC in the short-term potentiation of membrane resealing, ATP or AMP (100 µM) was applied to non-wounded cells, and membrane resealing was assessed 5–20 min after nucleotide application (Fig. 6). ATP potentiated membrane resealing, with resealing rates of ATP or AMP-treated cells of 0.052±0.0034 (*n*=35) and 0.031±0.0029 (*n*=14), respectively. In contrast, ATP did not potentiate cell membrane resealing in cells pretreated for 10 min with either Rp-8-Br-cAMPS (30 µM) or bisindolylmaleimide I (1 µM), with resulting resealing rates of 0.031±0.0029 (*n*=21) and 0.029±0.0028 (*n*=26), respectively. These results suggest that stimulation of PKA and PKC by ATP is required for short-term potentiation of membrane resealing in neighboring MDCK cells.

## DISCUSSION

Plasma membrane disruption evokes a rapid exocytic response that is required for membrane resealing in all cell types tested (McNeil and Steinhardt, 2003). It has been shown that repeated membrane disruptions within a single cell reseal faster than the initial wound, and this process occurs in a PKA- and PKC-dependent manner (Togo et al., 1999, 2003; Togo, 2004). Furthermore, approximately twice as much exocytosis was observed in second wounds compared with initial wounds, even though more rapid resealing of the second disruption reduced the Ca^2+^ influx (Togo et al., 2003; Togo, 2004). A previous study (Togo, 2014) and the present study have demonstrated that cell membrane disruption stimulates both cAMP and Ca^2+^ signaling in an ATP-dependent manner in neighboring MDCK cells. Furthermore, the present study suggests that PKA and PKC potentiate cell membrane resealing in neighboring cells in addition to wounded cells. These results strongly suggest that proteins involved in exocytosis are phosphorylated by protein kinases such as PKA and PKC, as has been observed in neurons (Shimazaki et al., 1996; Risinger and Bennett, 1999; Matveeva et al., 2001; Nagy et al., 2002, 2004), although the proteins phosphorylated upon cell membrane disruption have not yet been elucidated in either wounded or neighboring cells. These kinases are also known to regulate exocytosis in other cells types including pancreatic β-cells (Wan et al., 2004) and epithelial cells (Koh et al., 2000).

The present study revealed the spatiotemporal dynamics of cAMP signaling upon cell membrane disruption in MDCK cells. In neighboring cells, cAMP initially decreased and then increased transiently in an ATP-dependent manner (Fig. 2B). Furthermore, cell membrane disruption altered the baseline value of cADDis in neighboring cells in an ATP-dependent manner (compare c and c′ in Fig. 2B). The AC isoforms are regulated by Ca^2+^ signaling pathways, both positively and negatively, in addition to G proteins (Halls and Cooper, 2011, 2017). In fact, Ca^2+^ transients in neighboring cells occur 0–30 s after cell membrane disruption (Fig. 4), suggesting that Ca^2+^ signaling pathways affect the activity of ACs in neighboring cells. Furthermore, nitric oxide signaling, which is stimulated by cell membrane disruption (Togo, 2012), can also modulate some AC isoforms (Klein, 2002). Additional investigations, including identification of the AC isoforms expressed in MDCK cells, will reveal the effect of signaling pathways on the activity of ACs in neighboring cells upon cell membrane disruption.

As shown in Fig. 3, ATP treatment induced a prolonged increase in cAMP, whereas cell membrane disruption induced a transient increase in cAMP in neighboring cells. This is possibly due to release of ATP from wounded cells in a temporal event, because cell membrane disruption usually reseals in the presence of extracellular Ca^2+^ (Steinhardt et al., 1994).

Extracellular ATP mediates its effects through either P2Y G-protein-coupled receptors or P2X ligand-gated ion channels (Burnstock, 2007). Ca^2+^ imaging demonstrated that an increase in [Ca^2+^]_i_ in neighboring cells occurred even in Ca^2+^-free conditions (Fig. 4A), suggesting that most of the Ca^2+^ was mobilized from intracellular stores in neighboring cells upon cell membrane disruption. Thus, ATP released from wounded cells stimulates G proteins and, subsequently, phospholipase C via P2Y receptors in neighboring cells. In addition, the increase in [Ca^2+^]_i_ was slightly delayed under Ca^2+^-free conditions (Fig. 4B), suggesting that the sharper rise in [Ca^2+^]_i_ observed in the 1.8 mM Ca^2+^ condition arose from Ca^2+^ influx through P2X receptors. The identity of the P2 receptors involved in the short-term potentiation of membrane resealing in neighboring MDCK cells has not yet been identified. This will be the focus of further studies.

As shown in Fig. 4A, a prolonged increase in [Ca^2+^]_i_ in neighboring cells was observed upon cell membrane disruption under Ca^2+^-free conditions. This possibly arises from the continuous diffusion of ATP from wounded cells, as cell membrane resealing does not occur in Ca^2+^-free conditions (Steinhardt et al., 1994).

The present study clearly demonstrated that ATP plays an important role in the potentiation of membrane resealing via a paracrine mechanism. In addition, it is possible that autocrine signaling modulates membrane resealing responses in the wounded cell itself. The autocrine effects of ATP upon cell membrane disruption are currently under investigation.

## MATERIALS AND METHODS

### Cell culture

MDCK (NBL-2) cells (JCRB9029) were obtained from the Health Science Research Resources Bank of Japan Health Sciences Foundation, and cultured as previously described (Togo, 2014).

### Quantification of cAMP

Cells plated on six-well plates were used in assays at 100% confluency. Prior to treatment, the culture medium was removed and cells were incubated in 1.8 mM Ca^2+^ Ringer's solution containing 1% fetal bovine serum (FBS) and 100 µM 3-isobutyl-1-methylxanthine (IBMX), a phosphodiesterase inhibitor. Cells were wounded by scratching the monolayer with a 27G needle 20 times, or treated with 100 µM ATP or 100 µM forskolin. Two minutes after scratching or treatment, cells were washed once with phosphate-buffered saline and lysed with Sample Diluent buffer from a DetectX Direct Cyclic AMP Enzyme Immunoassay Kit (Arbor Assays, Ann Arbor, MI, USA). The amount of cAMP in each sample was subsequently determined according to the manufacturer's protocol, and normalized to the amount of total protein as determined by Qubit Protein Assay Kit (Thermo Fisher Scientific).

### cAMP imaging

The cADDis cAMP assay kit (#U0200G) was purchased from Montana Molecular (Bozeman, MT, USA). Transduction of the cADDis cAMP sensor was performed according to the manufacturer's protocol. Briefly, trypsinized cell suspensions were mixed with cADDis cAMP sensor BacMam stock and sodium butyrate, and plated on glass-based 35-mm dishes. The cells were incubated at room temperature for 30 min in the dark, and returned to normal culture conditions. After 24 h, the culture media were replaced with 1.8 mM Ca^2+^ Ringer's solution containing 1% FBS. Changes in fluorescence intensity in the cytoplasmic region were monitored with a LSM510 laser-scanning confocal microscope (Carl Zeiss, Oberkochen, Germany) equipped with an Axiovert (C-Apochromat 40×/1.2 W Corr objective). To disrupt the plasma membrane, glass needles were made from Narishige G-1000 glass rods by pulling with a Narishige PC-10. Wounding of cells was performed using an InjectMan 5179 and FemtoJet 5247 (Eppendorf, Hamburg, Germany) equipped with a microscope. The time setting for wound induction was 0.3 s.

### Ca^2+^ imaging

Cells plated on glass-based 35-mm dishes were incubated with 1 µM CG-1 AM (Thermo Fisher Scientific) at 37°C for 1 h. Culture media were then replaced with 1.8 mM Ca^2+^ Ringer's solution containing 1% FBS, and cells were observed with the same system as described for cAMP imaging.

### Membrane resealing assay

Cells plated on glass-based 35-mm dishes were loaded with 1 µM Calcein Red-Orange AM (Thermo Fisher Scientific) for 1 h at room temperature. Then, the cell membrane was disrupted with a glass needle in 1.8 mM Ca^2+^ Ringer's solution as described above, and the fluorescence of Calcein Red-Orange was monitored. Data acquisition was performed using the same system as described for cAMP imaging. A transient decrease in fluorescence intensity indicates successful resealing, whereas a persistent decrease in fluorescence intensity (as an indicator of dye loss) indicates resealing failure. The resealing rate was defined as the inverse of resealing time in seconds. For cells that failed to reseal, the rate was defined as zero.

### Statistical analysis

All values are shown as the mean±standard error of the mean. Statistical comparisons were performed using Prism 7 (GraphPad Software). A Mann–Whitney test was used to compare two groups. One-way ANOVA followed by Tukey's multiple comparison test was used to compare more than two groups. *P*<0.05 was taken to indicate statistically significant differences.

## Supplementary Material

Supplementary information
