# Supplementary material for: Cell membrane disruption stimulates cAMP and Ca2+ signaling to potentiate cell membrane resealing in neighboring cells
Source: Biol Open. 2017 Nov 1;6(12):1814–9. doi: 10.1242/bio.028977 (PMC5769656; doi:10.1242/bio.028977)
Supplement: Supplementary information [file biolopen-6-028977-s1.pdf]

Supplementary information

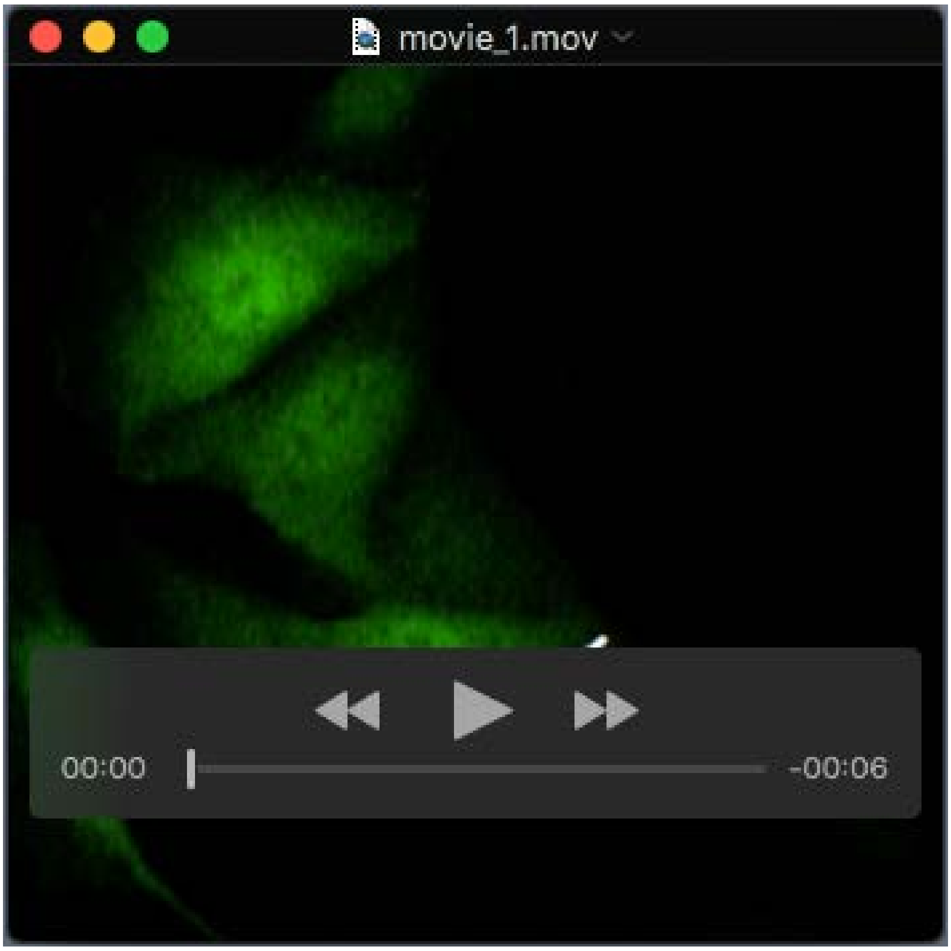

Movie 1. Spatiotemporal dynamics of cAMP upon cell membrane disruption visualized by Green Upward cADDis. The cell indicated by an arrow was wounded by a glass needle. The intensity of cADDis in the wounded cell decreased possibly due to efflux of cADDis. Frames every 2.5 s were used for the movie. The movie play rate is 30 frames/s.

Biology Open • Supplementary information

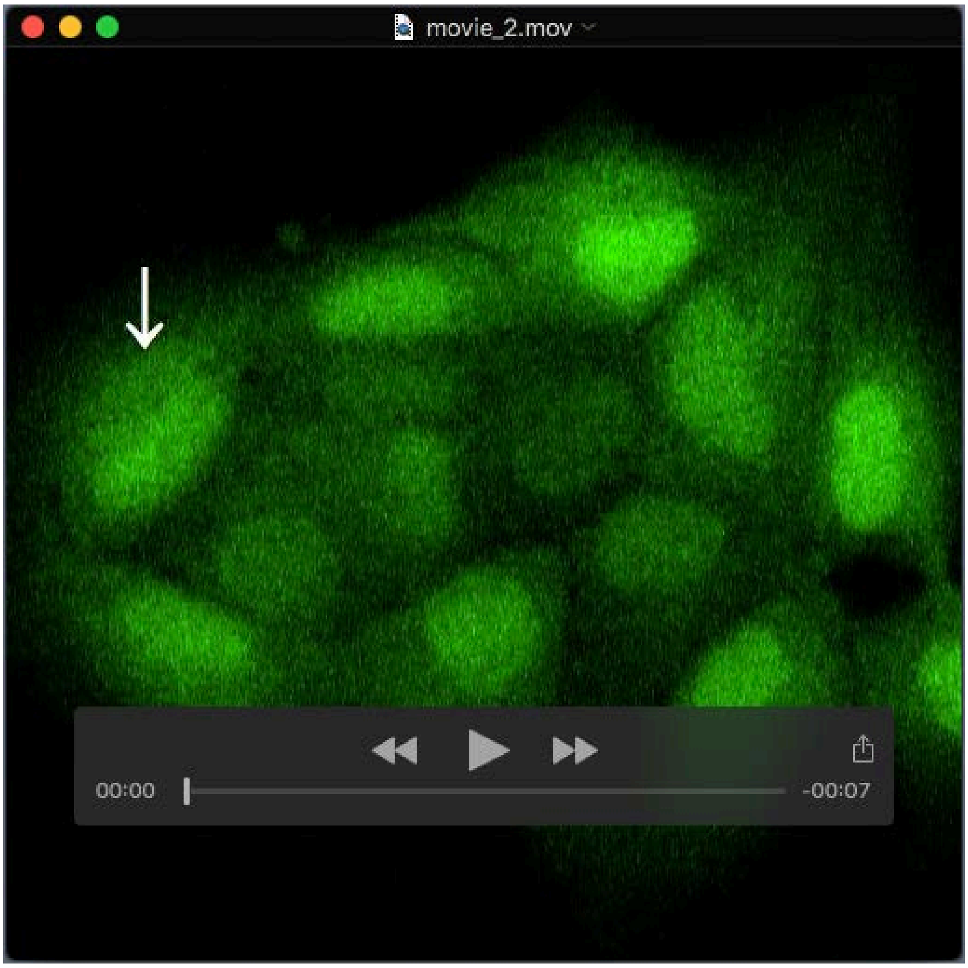

Movie 2. Intercellular Ca^{2+} wave upon cell membrane disruption. Cells loaded with Calcium Green-1 AM were wounded with a glass needle in the presence of extracellular Ca^{2+} (1.8 mM). The arrow indicates a wounded cell. The fluorescence changes in a wounded cell do not reflect the precise changes in $[Ca^{2+}]_i$ because cell membrane disruption results in efflux of Calcium Green-1. Frames every 3 s were used for the movie. The movie play rate is 10 frames/s.
